# Full-Thickness Skin Graft Versus Split-Thickness Skin Graft for Radial Forearm Free Flap Transfer in Oral Cavity Reconstruction: A Systematic Review and Meta-Analysis

**DOI:** 10.7759/cureus.49279

**Published:** 2023-11-23

**Authors:** Mohammad Saleki, Muhammad Ashhad Noor, Patrick Hurt, Ahmad Abul

**Affiliations:** 1 Medicine & Surgery, Queens Hospital, London, GBR; 2 Medicine & Surgery, Manchester University NHS Foundation Trust, Manchester, GBR; 3 Medicine & Surgery, Barts and The London School of Medicine, London, GBR; 4 Medicine, University of Leeds, Leeds, GBR

**Keywords:** ftsg, stsg, intra-oral tumour, full-thickness skin graft, split-thickness skin graft (stsg), craniofacial tumors, radial forearm free flap

## Abstract

The radial forearm free flap (RFFF) is a surgical technique for addressing intraoral reconstruction. However, with the limitation of an unavoidable defect at the RFFF donor site, split-thickness skin grafts (STSGs) have been a solution for repairing these defects, but they are not without challenges. This study aimed to evaluate an approach using full-thickness skin grafts (FTSGs), comparing their effectiveness in terms of aesthetics, pain, complications, and scarring. A systematic review and meta-analysis were conducted following the Preferred Reporting Items for Systematic Reviews and Meta-Analyses (PRISMA) guidelines. Studies comparing FTSG with STSG for RFFF donor site repair in head and neck cancer patients were included. Primary outcomes measured were appearance and pain at the RFFF site, and secondary outcomes were infection, tendon exposure, graft loss, and scar assessment. A meta-analysis and systematic review of eight studies demonstrated that FTSG provided a superior aesthetic appearance at the RFFF donor site compared to STSG (p = 0.001), with low heterogeneity among the studies. The analysis found no significant difference in donor site pain between techniques. There were no significant differences in infection, tendon exposure, or skin graft loss between the two graft methods. This study suggests that FTSG is comparable to STSG in terms of donor site pain, scarring, and infection while offering superior aesthetic outcomes.

## Introduction and background

The radial forearm free flap (RFFF) has long been a cornerstone in reconstructive surgery, particularly in the field of oral and oropharyngeal reconstruction, since its introduction in 1981 [1]. The RFFF is a surgical procedure that involves transferring skin, tissue, and blood vessels from the forearm to reconstruct areas in need. It is commonly used in head and neck surgeries [1,2]. Renowned for its attributes, including thinness, pliability, ease of re-innervation, and a remarkable success rate of approximately 90%-100%, the RFFF has played a pivotal role in addressing intraoral soft tissue defects [2,3]. However, a notable limitation has persisted in the form of an unavoidable defect at the RFFF donor site, precluding primary closure.

Split-thickness skin grafts (STSGs) have been the go-to solution for repairing RFFF donor site defects. Yet, this method presents its own set of challenges, including a secondary donor site and a high incidence of complications such as graft loss, delayed healing, tendon exposure, and suboptimal appearance [4,5]. Recognising these drawbacks, a variety of alternatives have emerged over the years, aiming to mitigate donor site morbidity while preserving aesthetics and function. These include full-thickness skin grafts (FTSGs) harvested from locations like the abdomen or groin and other innovative flap and graft techniques.

While some studies have explored the use of local FTSGs and compared them to STSGs in terms of subjective impairment and aesthetic outcomes, the adoption of an ipsilateral triangular FTSG to repair the RFFF donor site remains limited on the global stage [6,7]. Furthermore, no comprehensive study has undertaken a comparative analysis of both aesthetic and biomechanical outcomes of the hand following the repair of the RFFF donor site using an ipsilateral triangular FTSG in comparison to traditional STSG methods. 

In light of this gap in the existing literature, this meta-analysis endeavours to quantitatively assess hand function subsequent to the closure of RFFF donor sites, with specific attention to the innovative application of a triangle-shaped FTSG harvested in proximity to the donor site defect as opposed to the conventional STSG. By examining and synthesizing a wealth of empirical evidence, this study aims to shed light on the relative merits of these techniques, providing valuable insights for the advancement of reconstructive surgery and donor site management in the realm of RFFF procedures. We will compare the primary outcome measures like appearance and pain with secondary aims comparing complications including skin graft loss, infection, tendon exposure, and scar assessment.

## Review

Methods 

A systematic review and meta-analysis were conducted as per the Preferred Reporting Items for Systematic Reviews and Meta-Analyses (PRISMA) guidelines [8]. This study has been registered at the International Prospective Register of Systematic Reviews (PROSPERO) (registration number: CRD42023468825, https://www.crd.york.ac.uk/PROSPERO).

*Eligibility Criteria* 

All randomised control trials (RCTs) and observational studies comparing FTSG with STSG for patients undergoing RFFF for head and neck cancer were included. The FTSG was the intervention group of interest, and the STSG was the comparator.

Primary Outcomes 

The primary outcomes were the appearance and pain of the RFFF site, which were assessed using different scales, the most common being the visual analogue scale (VAS).

Secondary Outcomes 

Complications were reported as important secondary outcomes. The included studies had several reported complications; the ones included for meta-analysis were surgical site infection, tendon exposure, and skin graft loss at the donor site. Other secondary outcomes that were reported qualitatively were the grading of scars (ranging from poor to excellent) and scar vascularity and pigmentation, which were assessed using the Vancouver Scar Scale (VSS).

Literature Search Strategy 

Two authors, MS and AA, independently searched the following electronic databases: Medical Literature Analysis and Retrieval System Online (MEDLINE), Excerpta Medica Database (EMBASE), Cumulative Index to Nursing and Allied Health Literature (CINAHL), and the Cochrane Central Register of Controlled Trials (CENTRAL). The last search was run on September 30, 2023. Thesaurus headings, search operators, and limits in each of the above databases were adapted accordingly. In addition, the World Health Organization International Clinical Trials Registry (http://apps. who.int/trialsearch/), ClinicalTrials.gov (http://clinical- trials.gov/), and International Standard Randomised Controlled Trial Number (ISRCTN) Registry (http://www.isrctn. com/) were searched for details of ongoing and unpublished studies. No language restrictions were applied to our search strategies. The search terminologies included “full-thickness graft”, “split-thickness graft” and “(radial forearm flap) OR (forearm free flap)”. The bibliographic lists of relevant articles were also reviewed.

Selection of Studies   

The title and abstract of articles identified from the literature searches were assessed independently by two authors (MS and AA). The full texts of relevant reports were retrieved, and those articles that met the eligibility criteria of our review were selected. Any discrepancies in study selection were resolved by discussion between the authors. 

Data Extraction and Management

An electronic data extraction spreadsheet was created in line with Cochrane’s data collection form for intervention reviews. The spreadsheet was pilot-tested in randomly selected articles and adjusted accordingly. Our data extraction spreadsheet included study-related data (first author, year of publication, country of origin of the corresponding author, journal in which the study was published, study design, study size, clinical condition of the study participants, type of intervention, and comparison), baseline demographics of the included populations (age and gender), and primary and secondary outcome data. Authors MN and MS cooperatively collected and recorded the results, and any disagreements were solved via discussion.

Data Synthesis 

Data synthesis was conducted using the Review Manager ((RevMan) (Computer program) Version 5.4.1: The Cochrane Collaboration, 2020). The extracted data were entered into Review Manager by two independent authors, AA and MS. The analysis involved was based on the random effect model to account for heterogeneity. The results were reported in forest plots with 95% confidence intervals (CIs).

For dichotomous outcomes, the odds ratio (OR) was calculated between the two groups. The OR is the odds of an event in the FTSG group compared with the STSG group. An OR of greater than one for complications reported would favour the FTSG group, an OR of less than one would favour the STSG group, and an OR of one would favour neither group.

For continuous outcomes, the standardised mean difference (SMD) was calculated between the two groups (standardised was used due to the variation of scales between studies). A positive SMD for appearance and pain would favour the FTSG group, a negative SMD would favour the STSG group, and SMD of 0 would favour neither up.

Assessment of Heterogeneity   

Heterogeneity among the studies was assessed using the Cochran's Q test (χ2). Inconsistency was quantified by calculating I2 and interpreted using the following guide: 0% to 25% may represent low heterogeneity, 25% to 75% may represent moderate heterogeneity, and 75% to 100% may represent high heterogeneity. 

Results 

*Literature Search Results * 

**Figure 1:**
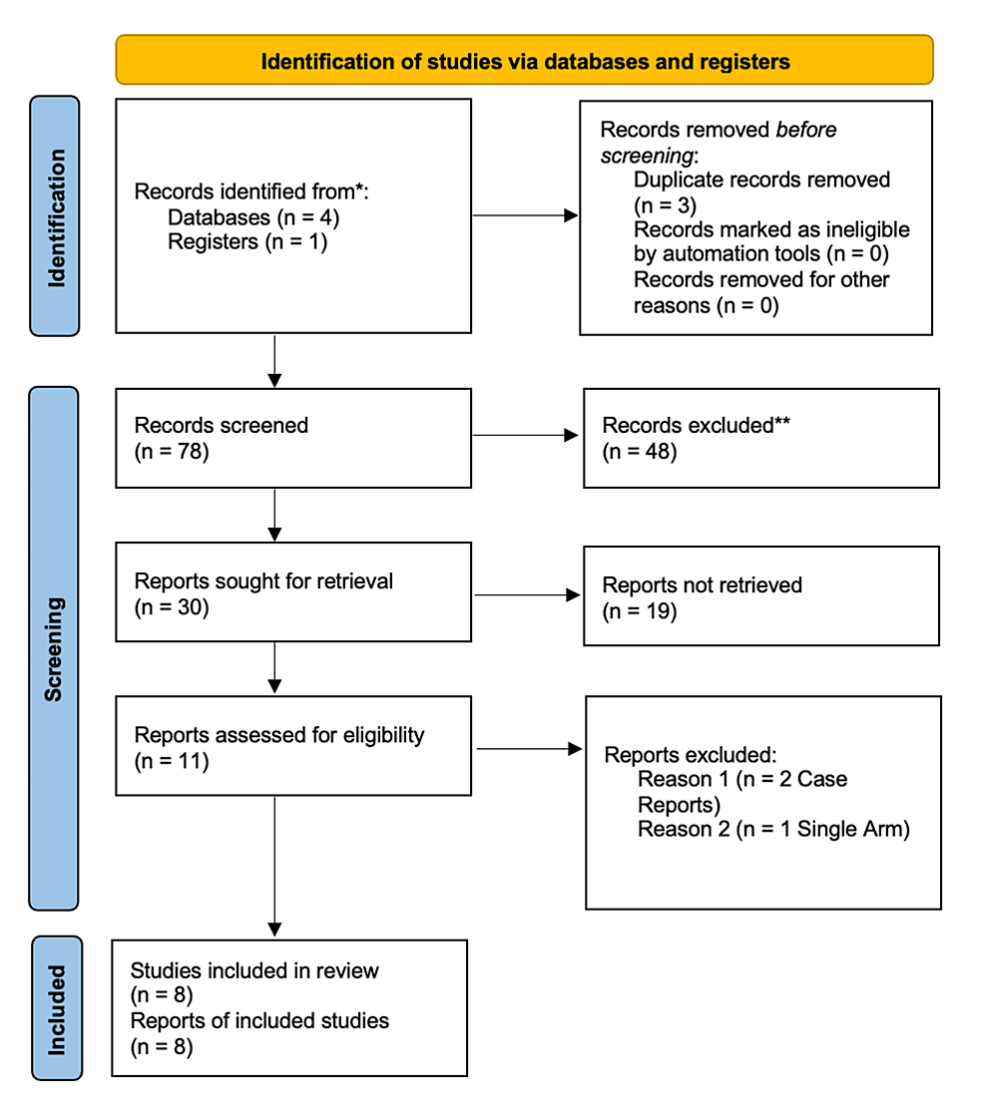
PRISMA flow diagram The PRISMA diagram details the search and selection processes applied during the overview. PRISMA: Preferred Reporting Items for Systematic Reviews and Meta-Analyses

Our search strategy retrieved 78 studies, and after a thorough screening of the retrieved articles, the authors identified eight studies that met the eligibility criteria (Figure 1).

Eight studies were identified as eligible for inclusion in this study [9-16].

Overview of Studies 

Chambers et al. [10] conducted a study presenting early experiences with the ipsilateral full-thickness forearm skin grafting (IFTFSG) technique for addressing radial forearm flap donor site defects, comparing its outcomes to split skin grafts. The study included data from 21 consecutive patients who underwent the IFTFSG technique. Patients were assessed preoperatively, and the non-dominant forearm was preferred for grafting. The study evaluated subjective patient opinions on altered sensation, pain, grip strength, and aesthetic results, as well as objective assessments of scar appearance. 

A prospective RCT by Sidebottom et al. [14] aimed to compare the outcomes of full-thickness and partial-thickness skin graft repairs for the radial donor site in patients undergoing RFFF reconstruction for head and neck malignant defects. Over an 18-month period, 68 patients were randomly assigned to receive either full-thickness or partial-thickness grafts. The study found that both groups had similar outcomes in terms of graft take at the radial recipient site, with an average of 93.4% success. There were no significant differences in the number of dressings or complications at the recipient site between the two groups. However, the FTSG group required fewer dressings at the donor site, indicating reduced morbidity. Additionally, there were no significant differences in patient-assessed aesthetic appearance or pain at either the recipient or donor sites. The study concluded that both graft types yield comparable short-term and long-term outcomes for RFFF site repair.

Between January 2001 and June 2004, Zuidam et al. [16] conducted a retrospective follow-up study to compare outcomes between two methods of donor site closure for RFFF in 34 head and neck cancer patients. Among these patients, 15 underwent STSG closure, while 19 underwent local FTSG closure. The study aimed to assess both subjective and objective outcomes, encompassing functional parameters, sensory perception, aesthetic results, and complications associated with each closure technique. Importantly, the study found no statistically significant differences between the two methods, indicating that V-Y local donor site closure with FTSG is a valuable technique for preventing additional donor site scarring and discomfort related to STSG closure. 

Davis et al. [11] conducted a retrospective chart review study aimed at assessing the functional and aesthetic outcomes of using either FTSGs or STSGs to cover the donor sites of RFFF reconstructions. The study included data from 47 patients who underwent radial forearm flap reconstruction between May 1997 and August 2004. Comparisons were made between patients who received STSG and those who received FTSG. Key outcomes measured included the number of postoperative dressings, incidence of tendon exposure, time to healing at the skin graft donor site, time to healing at the skin graft recipient site, and patient-reported aesthetic satisfaction and pain levels. The study found no statistically significant differences in terms of tendon exposure, time to healing at donor and recipient sites, or postoperative pain. However, patients who received FTSG reported higher scores for radial forearm scar aesthetics and satisfaction. Overall, the study suggested that FTSG coverage results in superior aesthetic outcomes for radial forearm flap donor sites without significant differences in functional outcomes. 

Krane et al. [12] carried out a retrospective case series aiming to compare the morbidity and aesthetic outcomes of FTSGs and STSGs in RFFF donor sites. Sixty-eight patients who underwent FTSG reconstruction were compared to 68 patients who had STSG reconstruction. The study found no significant differences in complications, including graft loss, tendon exposure, infection, paraesthesia, or hematoma/seroma, between the two groups. However, aesthetic outcomes were superior in the FTSG group, as assessed by both patients and surgeons. This suggests that FTSGs may provide better cosmetic results without additional donor site morbidity or wound creation compared to STSGs.

Peters et al. [13] conducted a retrospective study aiming to assess the aesthetic outcomes of radial forearm flap donor sites covered with either STSGs or FTSGs using objective measurement methods. The study involved 30 patients (15 FTSG, 15 STSG) whose forearms were scanned with a three-dimensional (3D) scanner. The surface differences between the original forearm and the filled area of the transplant were measured. The FTSG group showed a significantly lower surface difference compared to the STSG group. However, there were no significant correlations between surface deviation and the patient's subjective aesthetic satisfaction. Questionnaire results did not show significant differences between the two groups. The study concluded that both FTSGs and STSGs yield good aesthetic outcomes for RFFF donor site closure, with the 3D scanner providing an objective measurement method. 

A retrospective clinical study by Vahldieck et al. [15] aimed to compare outcomes between two groups of patients who had undergone RFFF donor site closure for head and neck malignant defect reconstruction. The study included 44 patients, with 19 patients in the STSG group (Group 1) and 21 patients in the local ipsilateral FTSG group (Group 2). The key outcomes assessed included functional parameters such as range of motion, grip strength, and skin sensitivity, as well as aesthetic outcomes evaluated using the Patient and Observer Scar Assessment Scale (POSAS). Patient satisfaction, including complaints related to activities of daily life and cold sensitivity, was also measured. The study concluded that FTSG from the forearm yielded better aesthetic results and fewer wristwatch-related complaints compared to STSG, while functional outcomes and cold sensitivity were similar between the groups. 

Al-Aroomi et al. [9] conducted a retrospective study to evaluate the functional and aesthetic outcomes following the closure of RFFF donor sites using FTSG vs. STSG techniques. The study included a total of 75 patients who underwent oral cavity reconstruction with RFFF between March 2017 and August 2021. These patients were divided into two groups: the FTSG group (n = 35) and the STSG group (n = 40). The primary outcomes measured included biomechanical grip strength, pinch strength, range of wrist movements, subjective donor site morbidity, and aesthetic and functional results. The results showed that the STSG group had better grip strength and wrist extension compared to the FTSG group. However, the FTSG group had advantages in terms of harvesting time and donor site appearance. Cold intolerance was more common in the STSG group. Overall, the FTSG group demonstrated better cosmesis and avoided additional donor sites, with minimal differences in hand biomechanics.

Baseline Characteristics Table 

The baseline characteristics of the included studies are summarised in Table 1.

**Table 1:** Baseline characteristics of the included studies NR: not reported

Study (Year)	Journal, Country	Study Design	Populations Involved	Dates Done	Age	Total Sample (Intervention : Control)	Interventions Compared
Al-Aroomi et al. (2023) [9]	International Journal of Oral & Maxillofacial Surgery, Denmark	Retrospective clinical study	Oral/Oropharynx Cancer	March 2017 - August 2021	Intervention: 54.6 ± 9.9 years (mean ± SD) Control: 56.7 ± 9.5 years (mean ± SD)	75 (35:40)	Traditional split-thickness skin graft versus full-thickness skin graft
Chambers et al. (1997) [10]	Journal of Cranio-Maxillofacial Surgery, Scotland	Retrospective clinical study	Major ablative head & neck surgery	NR	NR	21 (16:5)	Traditional split-thickness skin graft versus full-thickness skin graft
Davies et al. (2011) [11]	Journal of Hand and Microsurgery, India	Retrospective clinical study	Radial forearm reconstructions	May 1997 - August 2004	55.2 ± 15.6 years (mean ± SD)	18 (7:11)	Traditional split-thickness skin graft versus full-thickness skin graft
Krane et al. (2020) [12]	American Academy of Otolaryngology-Head and Neck Surgery, United States of America	Retrospective chart review	NR	April 2016 - November 2017	Intervention: 63.0 ± 15 years (mean ± SD) Control: 65.9 ± 10 years (mean ± SD)	136 (68:68)	Traditional split-thickness skin graft versus full-thickness skin graft
Peters et al. (2021) [13]	Journal of Craniofacial Surgery, United States of America	Retrospective clinical study	Oral/Oropharynx Cancer	NR	Intervention: 64.8 years (mean) 44-80 years (range) Control: 64.8 years (mean) 38-80 years (range)	30 (15:15)	Traditional split-thickness skin graft versus full-thickness skin graft
Sidebottom et al. (2000) [14]	International Journal of Oral & Maxillofacial Surgery, Denmark	RCT	Oral/Oropharynx Cancer	June 1996 - December 1997	NR	64 (32:32)	Traditional split-thickness skin graft versus full-thickness skin graft
Vahldieck et al. (2022) [15]	Journal of Cranio-Maxillofacial Surgery, Scotland	Retrospective clinical study	Oral/Oropharynx Cancer	January 2020 - January 2021	Intervention: 65 ± 18 years (mean ± SD) Control: 60 ± 15.25 years (mean ± SD)	40 (21:19)	Traditional split-thickness skin graft versus full-thickness skin graft
Zuidam et al. (2005) [16]	Annals of Plastic Surgery, United States of America	Retrospective clinical study	Oral/Oropharynx Cancer	January 2001 - June 2004	Intervention: 56.8 ± 10.2 years (mean ± SD) Control: 62.0 ± 9.0 years (mean ± SD)	34 (19:15)	Traditional split-thickness skin graft versus full-thickness skin graft

Bias

Overall, all studies included were shown to be of good quality based on the classification system used by the Newcastle-Ottawa Scale (NOS) (Tables 2, 3).

**Table 2:** Assessment of risk of bias for randomised trials using the Cochrane Collaboration tool FTSG: full-thickness skin graft; STSG: split-thickness skin graft

Author (Year)	Bias	Authors’ Judgement	Support for Judgement
Sidebottom et al. (2000) [14]	Random sequence generation (selection bias)	Low-risk	Patients were randomly allocated to receive either FTSG or STSG
	Allocation concealment (selection bias)	Unclear risk	No information was given regarding the randomisation technique
	Blinding of participants and personnel (performance bias)	High-risk	Blinding of the participants was not possible due to the nature of the study
	Blinding of outcome assessment (detection bias)	Unclear risk	No information given
	Incomplete outcome data (attrition bias)	Low-risk	Consistency in numbers reported by the study and no missing data
	Selective reporting (reporting bias)	Low-risk	All outcomes have been reported
	Other bias	Low-risk	Similar baseline characteristics in both groups

**Table 3:** Newcastle-Ottawa scale (NOS) to assess the quality of non-randomised studies

Study	Selection	Comparability	Outcome/Exposure
Al-Aroomi et al. (2023) [9]	****	*	**
Zuidam et al. (1997) [16]	****	**	***
Vahldieck et al. (2022) [15]	****	*	**
Chambers et al. (1997) [10]	***	*	*
Davies et al. (2011) [11]	****	*	***
Krane et al. (2020) [12]	****	*	***
Peter et al. (2021) [13]	****	*	***

*Primary Outcome Results* 

**Figure 2:**
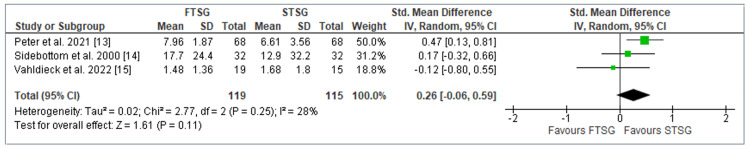
Forest plot of FTSG vs. STSG: Pain Analysis of pain outcomes using standardised mean difference The three studies included were Peter et al. [13], Sidebottom et al. [14], and Vahldieck et al. [15]. FTSG: full-thickness skin graft; STSG: split-thickness skin graft

Pain: Pain at the donor site was established as an essential outcome measure across six studies in our meta-analysis [10,11,13-16]. Among these studies, Peter et al. [13], Sidebottom et al. [14], and Vahldieck et al. [15] conducted quantitative assessments of pain, involving a total of 234 patients, and were thus included in the meta-analysis. Given the use of both VAS and numeric rating score (NRS) in pain measurement scales employed across these studies, we computed the SMD as a means of harmonising the data. Our analysis revealed no statistically significant distinction between the FTSG and STSG groups, indicating that both techniques were associated with comparable levels of pain at the donor site (SMD = 0.26; CI = -0.06 to 0.59; p = 0.11). Notably, our assessment of heterogeneity revealed a minimal degree of variability among the included studies (I^2 = 28%; p = 0.25) as depicted in Figure 2.

The remaining three studies (Zuidam et al. [16], Chambers et al. [10], and Davies et al. [11]) were subjected to qualitative analysis due to the distinct analytical methodologies employed in each investigation. Zuidam et al. [16] reported favourable pain outcomes in the intervention group when compared to the control group. Specifically, they reported donor site pain in only one instance within the FTSG group, in contrast to six instances within the STSG group. Similarly, Chambers et al.'s [10] findings indicated that moderate pain was experienced by only one FTSG patient, whereas three STSG patients reported such discomfort. In contrast, Davies et al.'s [11] results demonstrated mean pain scores that favoured the control group, with a reported mean pain score of 1.182 in the intervention group compared to 1.000 in the control group.

**Figure 3:**
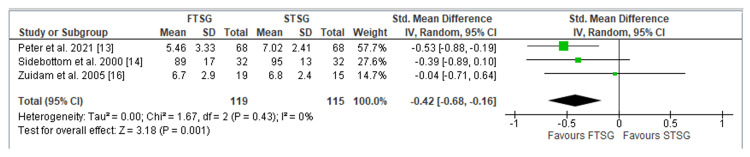
Forest plot of FTSG vs. STSG: Appearance Analysis of appearance outcomes using standardised mean difference The three studies included were Peter et al. [13], Sidebottom et al. [14], and Zuidam et al. [16]. FTSG: full-thickness skin graft; STSG: split-thickness skin graft

Appearance: The evaluation of graft site appearance, a critical parameter examined across three studies encompassing a total of 234 patients [13,14,16], produced the following results: the FTSG technique yielded a greater aesthetic appearance of the graft site as reported by patients (SMD = -0.42; CI = -0.68 to -0.16; p = 0.001). Importantly, our assessment of heterogeneity demonstrated minimal variability among these studies (I^2 = 0%; p = 0.43), as illustrated in Figure 3. 

*Secondary Outcome Results * 

Complications: Various complications were systematically assessed and compared across the included studies, with a particular focus on three commonly examined complications: infection, tendon exposure, and donor skin graft loss.

**Figure 4:**
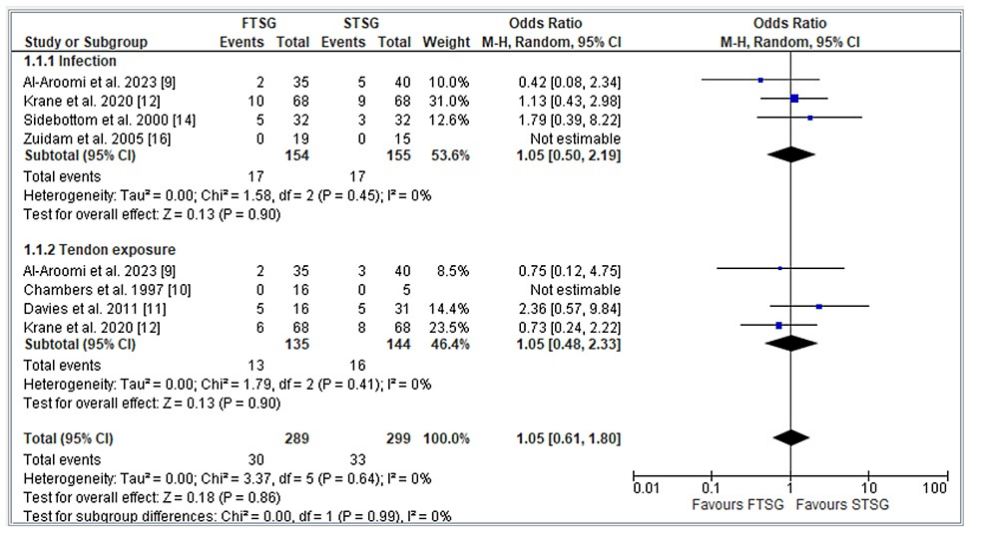
Forest plot of FTSG vs. STSG: Complications Analysis of complications including infection and tendon exposure using odds ratio. Studies included: Al-Aroomi et al. [9], Chambers et al. [10], Davies et al. [11], Krane et al. [12], Sidebottom et al. [14], and Zuidam et al. [16]. FTSG: full-thickness skin graft; STSG: split-thickness skin graft

Infection rates at the recipient site were documented in four studies [9,12,14,16], involving a total of 309 patients. Our analysis revealed no statistically significant difference in infection rates between the FTSG and STSG groups (OR = 1.05; CI = 0.48 to 2.33; p = 0.90). Importantly, our assessment of heterogeneity indicated minimal variability among these studies (I^2 = 0; p = 0.45), as illustrated in Figure 4.

Tendon exposure at the recipient site was reported in four studies [9-12], encompassing a total of 279 patients. Our findings demonstrated no statistically significant disparity in tendon exposure rates between the FTSG and STSG methods (OR = 1.05; CI = 0.61 to 1.80; p = 0.64). Consistently, our analysis of heterogeneity indicated minimal variability across these four studies (I^2 = 0; p = 0.64), as presented in Figure 4.

**Figure 5:**
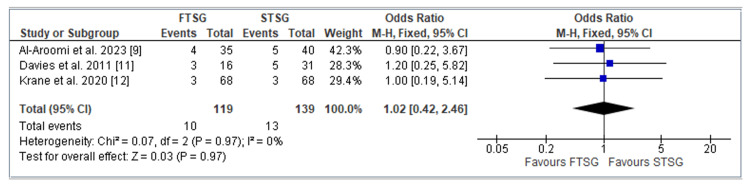
Forest plot of FTSG vs. STSG: Skin graft loss Analysis of the number of skin grafts lost using odds ratio. The three studies included were Al-Aroomi et al. [9], Davies et al. [11], and Krane et al. [12]. FTSG: full-thickness skin graft; STSG: split-thickness skin graft

The incidence of donor skin graft loss was assessed in three studies, specifically by Al-Aroomi et al. [9], Davies et al. [11], and Krane et al. [12], involving a total of 258 patients. Our analysis revealed no statistically significant difference in the rates of skin graft loss between the two groups (OR = 1.02; CI = 0.42 to 2.46; p = 0.97). Similar to the previous outcomes, our evaluation of heterogeneity indicated minimal variability within these three studies (I^2 = 0%; p = 0.97), as shown in Figure 5. 

Scarring: Scarring at the donor site was comprehensively assessed across three studies [10,11,13]. Due to the disparate methodologies employed by each study, quantitative analysis was not feasible.

Chambers et al. [10] and Davies et al. [11] both reported on the differences in patients' self-reported scar satisfaction when comparing the FTSG and STSG methods. The grading of scars was categorised as excellent, satisfactory, or poor. Chambers et al.'s [10] findings favoured the STSG method, with eight patients reporting an excellent scar outcome compared to three in the FTSG intervention group. In contrast, Davies et al.'s [11] results indicated a preference for the FTSG group, with excellent grading observed in five cases compared to one in the STSG group concerning scarring satisfaction.

Peters et al. [13], in contrast, assessed scarring outcomes using the VSS [17], where a higher score indicates a worse scarring outcome. Their analysis did not reveal any observable difference between the two groups, with scores of 6.03 ± 2.74 in the FTSG group and 5.53 ± 3.15 in the STSG group.

Discussion 

This study is the first meta-analysis and systematic review to evaluate the effectiveness of STSGs versus FTSGs in repairing RFFF donor site defects. The results of this study indicate that FTSGs are equivalent to STSGs in terms of donor site pain, scarring, and the incidence of complications, including infection, tendon exposure, and donor skin graft loss. The findings also demonstrate that FTSG is superior to STSG in terms of qualitative aesthetic outcomes. This information is of significant value to surgeons, equipping them with essential data for informed decision-making and enhancing patient counselling in cases requiring RFFF procedures.

The primary outcomes of the current study were aesthetics and pain. Our findings showed that FTSG provides a superior aesthetic appearance at the donor site compared to the control group (p = 0.001). A standard mean difference for both FTSG and STSG reported low heterogenicity and minimal variation between the studies, giving the results reliability. Variable follow-up periods were observed (six to 12 months). However, even the shortest six-month follow-up [13] remains sufficient for complete donor site healing, thus validating the results [18]. Additionally, our analysis found a non-significant SMD in donor site pain between FTSG and STSG. Heterogeneity analysis indicated minimal variation among the studies. These results align with the findings of Zuidam et al. [16] and Chambers et al. [10], who reported positive pain outcomes favouring FTSG in ratios of 1:6 and 1:3, respectively. However, Davis et al. [11] reported outlier results favouring the control STSG group for donor site pain. Overall, our analysis suggests that FTSG and STSG result in comparable postoperative pain.

The secondary outcomes assessed were scarring and other complications. Regarding donor site scarring, three papers reported conflicting results [10,11,13]. Davis et al. [11] favoured FTSG (5:1), Chambers et al. [10] favoured STSG (8:3), and Peters et al. [13] found no statistically significant difference between the two groups. These inconsistent findings make drawing reliable conclusions about postoperative donor site scarring challenging. Moreover, FTSG demonstrated complication rates comparable to STSG. Infection rates were analysed in four studies [9,12,14,16], and our meta-analysis found no significant difference between the FTSG and STSG groups (p = 0.09). The analysis benefits from large cohort sizes and minimal result variation, enhancing its validity. Additionally, analysis of tendon exposure and graft site loss data from four [9-12] and three [9,11,12] studies, respectively, again revealed no statistical difference between FTSG and STSG (p = 0.64 and 0.97, respectively), with minimal data variability.

The STSGs are composed of the epidermis and a superficial part of the dermis, in contrast to FTSGs, which involve the entire dermis [19]. Common donor sites encompass the thighs, legs, abdomen, back, arms, forearms, and chest [20]. The STSGs were first employed in 1872, initially favouring rapid wound healing and minimal scar formation [21] with a thin epithelial layer (0.15-0.3 mm) [22]. Later techniques adopted intermediate-thickness (0.3-0.45mm) grafts with an appreciable amount of dermis to combine the benefits of both STSG and FTSG methods [23]. Some groups introduced three-quarter-thickness grafts with manual dermatomes, enhancing graft success rates and expanding donor site options [24]. The FTSGs, in contrast, were first described in 1875 [25] and later established in 1893 as a valuable alternative to STSGs by Fedor Krause, who demonstrated good outcomes in 21 patients where the original STSGs were unsuccessfully transplanted [21]. The FTSGs now find extensive use, particularly in facial and palmar burn reconstructions, yielding superior aesthetics and functionality [26]. These favourable aesthetic outcomes with FTSGs are in line with the findings of our analysis. Despite these advantages, STSGs remain the prevailing and standard choice for RFFF donor site repairs.

From the available literature and the results of our meta-analysis, it can be concluded that FTSG is largely equivalent to STSG. However, inherent differences in the two techniques practically open the door to differential usage according to surgeons’ preferences and patient factors.

The FTSGs, when used in a V-Y fashion, are typically limited in size to approximately one-third the distance from the elbow to the wrist crease [16]. Nevertheless, other groups have previously reported defects as large as 80 cm being resolved with FTSG. In contrast, STSGs are more versatile for larger defects, with Zuidam et al. [16] reporting a maximum defect size of 96 cm. In the context of RFFF defects, FTSGs, even with their limited size, are usually sufficient for repair, but this must still be taken into account with larger defects. Furthermore, FTSGs generally offer a closer match in skin colour, texture, and contractility, while STSGs are more susceptible to hyper/hypopigmentation, significantly affecting aesthetic appearance [26]. Additionally, both thick STSGs and FTSGs provide the necessary strength and viscoelastic properties for durability in mechanically stressed areas like the forearm, elbow, and wrist creases, making them ideal for RFFF reconstructions [27]. These distinctions may shed light on the factors contributing to the observed favourable outcomes with FTSGs in terms of aesthetics, aligning with our findings.

Additionally, Sidebottom et al. [14] reported a significant difference (p = 0.01) favouring FTSG in terms of reduced dressing usage. In contrast, Davis et al. [11] found a slight, non-significant preference for FTSG (p = 0.07). These findings are clinically relevant, guiding both surgeons and patients in RFFF procedure decisions. The FTSGs not only potentially offer superior aesthetic outcomes but also practical advantages, evident in reduced dressing requirements. This holds significant implications for decreasing patient discomfort during dressing changes, minimising stay time and unnecessary hospital visits, improving cost effectiveness, and enhancing the overall quality of life [28,29]. However, earlier research by Zuidam et al. [16] suggested a non-significant difference in hospital admission times, with the FTSG group experiencing a 3.6-day shorter duration (p = 0.06). These results challenge the notion that reduced dressing usage in FTSG equates to shorter hospital stays. Nonetheless, the other benefits of FTSG related to dressing changes remain relevant and may offer reasons to favour FTSG over STSG. In summary, additional high-quality evidence from robust RCTs with large cohorts is required to conclusively elucidate these potential advantages. There would also be a benefit in exploring differences in other factors such as length of admission, length of operation, function, and morbidity of the donor arm in future studies.

The inherent limitations of this review should, however, be taken into account when interpreting the results. Non-compatible analytical methods used by Davis et al. [11], Zuidam et al. [16], and Chambers et al. [10] made comprehensive meta-analysis of pain outcomes impossible and likely contributed to the variability of the findings observed. As such, each result lacks statistical power due to the relatively small population sizes in each, with the largest group being Krane et al. [12] (n = 136) followed by Al-Aroomi et al. [9] (n = 75), and the smallest group being Davies et al. [11], with only 18 participants. Therefore, these findings must be viewed with reservations. In addition, the studies involved in the analysis of scarring were constrained by drastic differences in the methodology of assessing scarring between the three groups, which inhibited meaningful meta-analysis. Davis et al. [11] and Chambers et al. [10] reported using variable qualitative scaring scores, while Peters et al. [13] incorporated a standardised VSS. Confounding this, individual results are limited by small n-numbers, thus reducing the power of each study individually. This highlighted a fundamental limitation of the current literature and, as a result, this meta-analysis. Moreover, these findings again demonstrate the need for a standardised method for assessing scarring to provide more meaningful data on scarring outcomes with FTSG versus STSG. The extreme variability in the reported scaring outcomes shown by Chambers et al. [10], Davies et al. [11], and Peters et al. [13] also highlight the problems associated with small cohort retrospective studies and emphasise the need for large prospective RCT studies to effectively evaluate the different modalities for repairing RFFF donor site defects and further the current evidence base.

## Conclusions

This meta-analysis suggests FTSG may provide better aesthetic outcomes with equivocal outcomes in terms of pain, scarring, and common complications compared to STSG for RFFF. The importance of these findings is that both methods can be used reliably according to the surgeon's preference, the size of the defect, and the match of the donor site skin. For further reliable conclusions, the current literature needs more high-quality RCTs with large cohorts, standardised qualitative measures, and analysis to further the current evidence base and allow for a more comprehensive meta-analysis of FTSG versus STSG.
